# First Experiences with *Online Last Aid Courses* for Public Palliative Care Education during the COVID-19 Pandemic

**DOI:** 10.3390/healthcare9020172

**Published:** 2021-02-05

**Authors:** Georg Bollig, Stefan Meyer, Boris Knopf, Marina Schmidt, Eithne Hayes Bauer

**Affiliations:** 1 Medical Research Unit, Institute of Regional Health Research, University of Southern Denmark, 5230 Odense, Denmark; EHB@rsyd.dk; 2 Palliative Care Team, Medical Department Sønderborg/Tønder, South Jutland Hospital, 6400 Sønderborg, Denmark; 3 Last Aid International, 24837 Schleswig, Germany; boris.knopf@letztehilfe.info; 4 Letzte Hilfe Deutschland gUG, 24837 Schleswig, Germany; stefan.meyer@letztehilfe.info (S.M.); marina.schmidt@letztehilfe.info (M.S.)

**Keywords:** death, dying, home death, palliative care, Last Aid course, online course, compassionate communities, COVID-19, pandemic

## Abstract

The Last Aid course aims to teach public palliative care by increasing public awareness and empowering people about the role of the individual in the death of loved ones. The Covid-19 pandemic, however, has altered educational methods prohibiting classroom settings. Therefore, an online course was created to enable continued and safe public palliative care education. A mixed-methods study was performed to examine the feasibility of delivering the Last Aid course online. Data collection included participant questionnaires with qualitative and quantitative data, observations and a focus group discussion. Data were analyzed using descriptive analysis and qualitative description. In total, 15 online Last Aid courses were held, 174 participants took part in the study and 92 completed questionnaires were included. Findings revealed overall course satisfaction for the online courses in line with previous findings for classroom teaching. The online platform enabled course participation from people previously unable or unwilling to attend, namely caregivers to dying relatives and younger people. Instructors displayed an ability to teach online. However, some instructors expressed frustration over reduced interaction and technical challenges, which was echoed by participant ratings showing that many lacked social networking with fellow participants. Nonetheless, this pilot study demonstrates the feasibility of the online Last Aid course. Attention must be given to increasing both participant-to-participant and instructor-to-participant interaction. More research on the long-term effects of Last Aid courses is needed.

## 1. Introduction and Background

Many people in different European countries would prefer to die at home [1]. Experts predict an increased need for palliative care and end-of-life care in the community in the future as a result of demographic changes and an increasing number of elderly people with multi-morbidity, dementia and frailty [2,3]. One of the most important public health issues for the future entails, therefore, the provision of palliative care in the community for all in need. As a major public health issue, Kellehear maintains that palliative care should be everyone’s business [4,5]. Relatives and general practitioners are considered central to palliative care provision in the home [6,7]. Thus, all people have a role of utmost importance in the provision of end-of-life care in the community setting. However, death-illiteracy is prevalent, most people lack knowledge about end-of-life care and a negative perception of palliative care in the public in general is apparent [8,9,10,11]. Public palliative care education (PPCE) has been neglected as a major public health issue for many years. One means of educating the public about palliative care is the Last Aid course (LAC). The idea of Last Aid courses and the public knowledge approach to palliative care were first described in 2008 [12]. Last Aid courses for the public were then introduced between 2014 and 2015 in Norway, Germany and Denmark in order to educate people about palliative care and to enhance the public discourse about death and dying in general [12]. LACs have spread to 17 countries across three continents—Europe, South America and Australia—and the courses have been welcomed and well accepted by people from various countries and cultures. A recent editorial highlighted the role of the Last Aid course for the development of personal skills, strengthening community action and proposed an annual World Last Aid Day [13].

The COVID-19 pandemic that has affected many countries and regions in the world from Spring 2020 prevented Last Aid courses for the public from being held in a standard classical classroom setting. Conversely, the pandemic also led to an urgent need for end-of-life care education of people and an increased public awareness of death, including a growing interest in talking about death and dying.

The aim of this pilot study was to test the feasibility of online Last Aid courses and to describe the first experiences of participants and instructors with the online last aid course format as a measure of teaching the public about palliative care, and providing a platform for discussing death and dying during the COVID-19 pandemic.

## 2. Materials and Methods

### 2.1. The Last Aid Course Concept

The Last Aid course is based on an international consensus of the International Last Aid working group [12,14]. The standardized course consists of 4 modules each of 45 min duration. Table 1 provides an overview of the Last Aid course contents. The course content of the Last Aid course includes end-of-life care, advance care planning and decision-making, symptom management, burial and cultural aspects of death and bereavement. Under normal circumstances, the course is held in a classroom setting with up to 20 participants within a timeframe of 3 to 4 h (including break) on a single day.

### 2.2. Last Aid Course Instructors

All Last Aid course instructors are people with practical experience within the field of palliative care including, e.g., nurses, doctors, social workers or volunteers. According to the international binding course rules, two certified instructors must hold the course together, one of whom must be a doctor or a nurse.

Instructors who held online Last Aid courses during the study period all possessed experience with Last Aid courses in the classroom setting and all received written guidelines on how to perform online Last Aid courses. The guideline was compiled in online meetings of the Online Last Aid working group of Letzte Hilfe Deutschland gUG, a German non-governmental organization.

### 2.3. Working Group for the Online Last Aid Course

The Online Last Aid working group of Letzte Hilfe Deutschland gUG was established in April 2020 in response to the challenges caused by the COVID-19 pandemic [15]. The first online LAC (OLAC) was held in April 2020 by two experienced Last Aid course instructors as the first proof-of-concept measure before the working group was created. Data from this course form part of the data of the present study. All registered German-speaking certified Last Aid course instructors were invited by email to participate in the working group. Overall, 34 certified Last Aid course instructors became members of the working group and made a commitment to work actively in the group and to provide online Last Aid courses for citizens. Between 11 April and 14 May, the working group used video meetings to approve a guideline for the online Last Aid course, including recommendations and rules for implementation. Members of the working group tested the online Last Aid course and provided the data for this article.

### 2.4. Setting and Participants

All German online Last Aid courses that were held from April 2020 to October 2020 and complied with the international Last Aid course rules were included in the study. Furthermore, data from the first two OLACs from Brazil were included. Course participants were asked to complete a questionnaire on their experience with the online course. In addition, Last Aid course instructors were asked to provide written comments of their experiences with the online course and to participate in an online focus group discussion.

### 2.5. Data Collection and Data Analysis

A questionnaire was used to evaluate the Last Aid course participants’ views on the online Last Aid course (Appendix A Table A1). The questionnaire was based on an existing Last Aid course questionnaire that is used to evaluate participants’ views directly after the course. It was adapted to include information about communication and interaction during the online course. The instructors asked course participants to return the questionnaires to them which they then forwarded together with their reports and comments to Letzte Hilfe Deutschland gUG. As different video platforms were used in the study, some informants could complete the questionnaire directly after the online meeting while connected to the online platform and others had to use a file or printout version of the questionnaire which they returned via Email.

The German instructors who taught OLAC were invited to participate in an online focus group discussion. The focus group discussion and evaluation took place during a video meeting of the Online Last Aid working group. The focus group discussion was led by two of the authors (M.S. and S.M.) while two other authors (G.B. and B.K.) were observers. For the focus group discussion, an open-ended interview technique was used with follow-up questions related to the informants’ statements. Opening questions used in the focus group discussion were:

- What are your experiences with the online Last Aid course?
- What are advantages or disadvantages of the online Last Aid course?
- Which differences did you experience when teaching online Last Aid courses compared with normal Last Aid courses?

During the meeting, notes were taken by two of the authors (M.S. and G.B.). In addition, the instructors were asked to provide written feedback on their experiences with the OLAC and their views on differences in comparison with the classical classroom based Last Aid Courses.

Inclusion criteria:

- OLAC was held online with two certified instructors.
- The OLAC questionnaire was used for the evaluation.

Exclusion criteria:

- Questionnaires that were received but could not be connected to an OLAC.
- Questionnaires that were incomplete.

The questionnaire for LAC was used instead of the OLAC questionnaire.

A mixed-methods design was used for the present study [16]. Data analysis was performed using both quantitative and qualitative methods. A mixed-methods approach was used in order to provide a rich picture of the personal experiences with the OLAC from the perspectives of both participants and instructors. Descriptive statistics were used to describe the quantitative data from the questionnaires. The qualitative data from the written feedback of both course participants and instructors were analyzed using qualitative description [17,18,19]. Qualitative description is a method that is well-suited to providing rich and straightforward descriptions of experiences or events in everyday terms and is especially useful in mixed-methods research [20]. Qualitative data were derived from the questionnaires from the course participants and written notes from the instructors and from the focus group discussion. Quotations from Last Aid course participants from the feedback directly after the online course and observations carried out by the instructors and authors were written down and used as field notes in qualitative data analysis [20]. Data analysis was based on qualitative description and qualitative content analysis using an inductive approach with data-derived themes [17,18,19,20]. The qualitative data from the questionnaires and written notes were read repeatedly by G.B. and M.S. and preliminary codes were established. Thereafter, the codes were discussed with the other co-authors and revised accordingly. G.B. and M.S. checked the data in order to question the findings. Meta-positions were used to critically reflect on and question our findings. All co-authors discussed the findings and agreed about the codes, themes and interpretation of the data.

In order to assess and ensure rigor of our findings, a combination of several strategies was used [20,21,22]. Purposeful sampling led to a choice of relevant informants with experience in the topic that enhances the transferability of the findings. All participants had the opportunity and were encouraged to speak or write freely about their experiences with the OLAC. Source triangulation was used in order to compare the views of participants and instructors from different OLACs from different regions aiming for a thick and rich description. The codes and themes that were found in the dataset by different authors were compared and discussed by the authors. Recurrent experiences and patterns were found from different OLACs and informants showing confirmability, recurrent patterning and confirmability of the findings.

### 2.6. Ethical Considerations and Ethics Approval

For the participants taking part in the study, completion of a questionnaire after participation in an OLAC was the only requirement. Therefore, no formal approval by an ethics committee was required. This study is part of an ongoing study to evaluate the effects of Last Aid courses which has been reported to the Regional Ethical Committee of Southern Denmark. The Regional Ethical Committee concluded that no formal application was required (The Regional Committees on Health Research Ethics for Southern Denmark; nr. 20182000-33). Informed consent was obtained from all participants who received information about the purpose of the study. In order to protect privacy, no personal data were collected.

## 3. Results

### 3.1. Results of the Work of the Online Last Aid Working Group

The result of the work of the Online Last Aid working group was a written guideline for online Last Aid courses that provides guidance in performing online courses. The recommendations included in the guidelines were tested in a trial period between April and October 2020. During a video meeting in August 2020, a definitive consensus on the OLAC guidelines was reached within the working group. The current guideline is a 10-page document that includes general recommendations and advice about the practical aspects of holding OLACs and experiences of the instructors with OLACs from the trial period. The guideline includes general recommendations for the course framework and suggestions for adaptation of the four Last Aid course modules concerning materials and methods that are slightly different from the guidelines for normal Last Aid courses known from the German Last Aid instructor handbook. In addition, the guideline includes advice from an experienced psychologist on how to deal with feelings and crying in group situations (online and offline) and instructions for the evaluation of the OLAC using the online Last Aid course questionnaire (Table A1). The guideline is available online for all certified Last Aid course instructors who want to implement online Last Aid courses. A short English summary of the guideline is provided as Supplementary Materials File S1. More information about the complete guideline is available via the first author on request.

### 3.2. Results of the Online Last Aid Course Questionnaire

**Figure A1:**
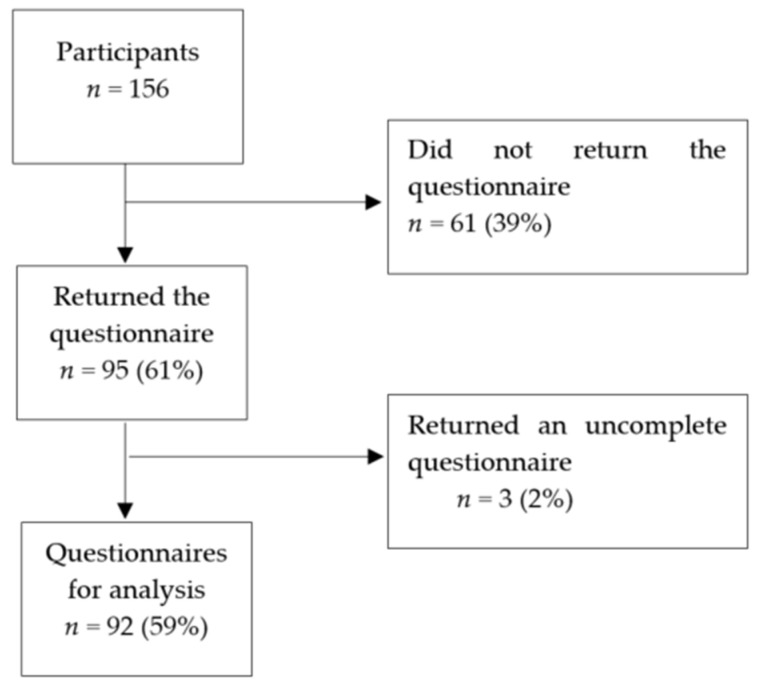
Flowchart of Last Aid course participants, respondents and included questionnaires.

During the study period between April 2020 and October 2020, a total number of 15 OLACs with a total number of 174 participants were registered (13 in Germany and 2 in Brazil). Two OLACs with 18 participants were excluded as a result of the use of an incorrect questionnaire. Thus, 156 online Last Aid course participants were included in the study and 92 completed questionnaires were eligible for analysis (Appendix B Figure A1).

The overall results from the questionnaire showed that the online Last Aid course is feasible and very well accepted by the participants. The quantitative data showed the following results: 80% of the respondents rated it as very good and 100% would recommend the course to others. The opportunity for participation in discussions was rated as very good by 44% of the participants whereas 18% found it satisfactory or enough. Table 2 and Table 3 summarize the ranking and answers from the questionnaire.

The qualitative data from the online Last Aid course questionnaire led to the formation of the following four themes. (1). Is an online Last Aid course only the second-best choice?; (2). meeting place with pleasant atmosphere; (3). helpful information and clear structure and (4). empowerment of participants.

#### 3.2.1. Is Online Last Aid Course Only the Second-Best Choice?

The participants appreciated the opportunity to receive information and to discuss death and dying online via video meetings especially during the current COVID-19 pandemic, but some would still prefer direct personal contact during a group meeting.

“I think online courses are an additional option to provide information and to enable communication with each other, but the direct personal contact still would be my preferred choice.”

“To me the online course is an emergency measure. Especially for the themes of death and dying personal contact is very important. I am looking forward to when normal courses can be held again.”

Although discussions are feasible online, the personal contact was described as limited and some participants missed private conversations in the breaks.

“The possibility for discussion and exchange is limited because talking together (in private) during the breaks is missing.”

Other participants experienced fruitful and meaningful discussions during the OLAC.

“I appreciated the extensive discussions.”

Some participants appreciated the opportunity for participating in the Last Aid course via video from home and recommended that this option should also be offered when the pandemic is over.

“Please continue the online format also when normal courses become available again.”

#### 3.2.2. Meeting Place with Pleasant Atmosphere

A number of participants described the positive and safe atmosphere that could be established during the online course.

“I found the atmosphere very pleasant and was astonished how well the course format works online.”

“An atmosphere of trust could be established although we were not sitting together in a meeting room.”

#### 3.2.3. Helpful information and clear structure

Participants welcomed the OLAC as a means of gaining information about palliative care and a meeting place to talk openly about death, dying and existential questions. They liked themes such as strategies to care for others, advanced care planning and final goodbyes.

“The course offers valuable and helpful information…It shows the options for support that are available.”

“The clear structure made it easy to participate…I appreciate the combination of information and open exchange very much.”

Participants experienced that questions were addressed and answered sufficiently.

“My important questions about caring for others were answered during the course.”

All participants stated they would recommend the OLAC to others and suggested that everybody should participate in Last Aid courses.

“Everyone should participate in a Last Aid Course.”

#### 3.2.4. Empowerment of Participants

Following participation in the OLAC many participants felt encouraged in engaging in end-of-life care and felt more confident about the importance of their personal contribution to palliative care provision in the community.

“The course contents helped me to feel a little more secure in dealing with dying and I feel that my inhibition in encountering dying people has become less.”

Many participants were reassured that they could ask for professional help and acquire knowledge about where to get support.

“I feel encouraged to ask for support and know now where to ask for it.”

### 3.3. Results from Reports, Field Notes and Comments of the Last Aid Course Instructors

Feedback from the OLAC instructors was received by both written reports and emails after the course, oral feedback during a video meeting of the working group and personal conversations with two of the authors (G.B. and M.S.).

#### 3.3.1. Online Last Aid Course Only the Second-Best Choice to in-Person Classes

The overall view of most Last Aid course instructors was that OLACs are feasible and meaningful in times of a pandemic where group meetings are restricted or impossible due to the social distancing strategy to prevent the spread of COVID-19. Most instructors had positive experiences with the OLAC. Nevertheless, most instructors stated that they would prefer physical group meetings for holding an LAC instead of OLAC in the post-pandemic time.

“For the future, we have planned to offer the Online Last Aid Course once a year…My personal view is that normal Last Aid Courses are much better.”

Some instructors found holding an online course challenging due to technical problems despite a pre-meeting with testing of the technical aspects usually being offered to the participants. In some cases, a third person was used to take care of the technical aspects. Some instructors strived to keep personal contact with the group and to scan the moods of the participants. Some instructors lacked more direct feedback from the group in comparison to the feedback possibilities that they are familiar with from normal LACs.

“In contrast to normal Last Aid Courses, it was more difficult to establish contact with the participants. For most of them (the participants) it was their first online course. I believe they were just a little insecure and were afraid of asking too much.”

In contrast to the above, some instructors stated that they found the OLAC more intense and explained that this may be due to the possibility that participants felt more comfortable when taking part from home.

Information about local options for palliative care support are a normal and essential part of the LAC. This proved to be more complicated in online courses because the participants came from different regions in Germany and sometimes from other countries too. Therefore, information could not be provided by the instructors on local palliative care support. Instead, instructors were limited to providing general advice on how to find palliative care options via internet sources. Some instructors solved this challenge by sending a list with links to the participants after the OLAC. A few offered follow up contacts via telephone for course participants. Many instructors described problems in relation to obtaining written feedback using the questionnaire from the OLAC participants.

#### 3.3.2. New Ways of Teaching Practical Last Aid

Some instructors reported sending a care package to participants before the course including samples of mouth care material and a scented cloth to demonstrate the use of aroma therapy, info material, etc.

“We sent mouth-care material and a scented cloth plus the evaluation questionnaire to the participants by normal mail.”

In order to illustrate practical Last Aid measures like mouth care, some instructors used a live demonstration during the online course, while others used a pre-recorded video.

#### 3.3.3. New Participant Groups

Some instructors recognized that there were other groups of people participating when compared to the usual participants in the OLAC. These included a higher number of younger people and people who were currently caring for seriously ill persons or persons dying at home. The fact that relatives who are carers are able to participate in the OLAC from home was rated as an advantage by many instructors, as this group of participants may otherwise be unable to attend a course.

“We were not astonished that the participants came from other groups than usual, especially many were of younger age.”

“Nearly all participants that came via the Last Aid homepage were actually providing care for someone, it was a really hot topic for them. Near all participants had relatives who were sick. That was a much higher number of caring relatives as usual in our Last Aid Courses.”

## 4. Discussion

The main findings from our explorative pilot study show that online Last Aid courses are feasible and welcomed by both participants and Last Aid course instructors. Although most participants consider the OLAC as just a second-best choice, some see advantages and recommend having OLAC courses as an option, also in times without restrictions for group meetings. Some instructors describe other groups such as younger people and caregivers were participating. The reasons for that are unclear. Possibly, people who care for others at home do not have an opportunity to be away from home because they are the only carer. Overall, 100% of Last Aid course participants would recommend the OLAC to others, 90% felt better prepared to encounter death and dying and 81% felt more prepared to care for dying people after the course. These findings indicate a huge potential to encourage people to participate in end-of-life care and are similar to results from a large study with more than 5000 LAC participants from Germany, Austria and Switzerland (manuscript under review).

Both participants and instructors describe reduced interaction and discussion in the OLAC. Interactivity is an important factor for internet and video-based education and must be taken into account in order to address the needs of the participants [23]. As mentioned in the introduction, palliative care should be everyone’s business but death-illiteracy is prevalent and most citizens lack knowledge about end-of-life care [4,5,8,9,10,11]. Therefore, it is interesting that the online Last Aid course, as a short educational video meeting, seems to strengthen the participants’ willingness to participate in end-of-life care. Hence, it may contribute to empowering the public in supporting dying people at home. However, willingness is not action and knowledge alone is not sufficient in order to change practice, as Ferris reminds us [24]. Therefore, further research including an evaluation of the long-term effects of LAC an OLAC on actual participation in end-of-life care using longitudinal studies of course participants is urgently needed. Last Aid courses may play an important role in a public health palliative care approach by developing peoples’ personal skills and community action [13].

The first experiences from this pilot study on OLAC and previous results from LAC [25] show that people like to talk about death and dying and want to learn how to provide basic palliative care at home. This can be achieved by the short Last Aid course with four teaching lessons and a minimum time consumption [12]. Furthermore, it has been shown that children and teenagers want to talk about death and to learn end-of-life care [25]. As described above, many instructors experienced problems in obtaining written feedback using the questionnaire from the OLAC participants after the course. This is in contrast to a response rate of 91% for the questionnaire-based evaluation from LAC (manuscript under review). This highlights a challenge that has to be taken into account in further research on both Last Aid and online Last Aid courses.

In summary, the experiences with both LAC and OLAC for citizens are very promising. OLACs are feasible, well accepted and can help to provide a knowledge basis for palliative care education for the public. They may contribute to preparing people for end-of-life situations and participating in palliative care at home. This is especially useful in situations such as the current COVID-19 pandemic. Public palliative care education via LAC and OLAC has considerable potential to inform the public and to encourage open discussions about death and dying. However, scientific evidence on the effects of Last Aid courses is limited and the current explorative pilot study has some shortcomings and biases as described below. The effects and the importance of LAC and OLAC in public palliative care education, therefore, warrants further investigation. A long-term follow-up on the individual level as well as looking at the bigger picture in terms of the public health impact and organizational aspects, including palliative care service provision at home and the number of home deaths should be performed. In addition, further investigation should explore the impact of cultural aspects for public palliative care education in general and the Last Aid course curriculum in particular.

### Future Directions

There is a need for further research on Online Last Aid Courses and Public Palliative Care Education. An international Last Aid Research Group Europe (LARGE) has been established with the aim of investigating the effects that Last Aid courses have on a personal, organizational and social level in the long term. The results from the current pilot study will inform future research on the topic. The European Association for Palliative Care (EAPC) has established a task force Last Aid and Public Palliative Care Education [26].

## 5. Limitations

The present pilot study has several limitations. A major limitation is the relatively small number of participants compared to a larger study on LACs (manuscript under review). The online Last Aid course questionnaire employed was developed and implemented within a short timeline due to the pressing need to establish online courses to address the publics’ needs during the COVID-19 pandemic. There are some bias issues concerning the used questionnaire [27] including not using a 5-point Likert-scale instead of the four options and not providing a third option in addition to yes or no answer opportunities. Future evaluation will take these possible biases into account and further develop the existing questionnaire based on recommendations for avoiding biases in questionnaires [27] and the experiences gained in this study. Another limitation is the lack of data about participants’ gender, age, occupation or their participation in palliative care. The decision not to collect personal data was made because many people were very concerned about providing personal information during the COVID-19 pandemic. A reason for this might have been the registration of data connected to visits in restaurants or meetings during the pandemic in Germany. Future studies should aim to address possible differences concerning these factors. At present, future studies to evaluate LAC and OLAC are in the planning phase. Although there are several limitations, the current pilot study is an important contribution to our knowledge as it shows the feasibility and acceptability of online Last Aid courses that may have a huge impact especially during pandemics.

## 6. Conclusions

The results of the current pilot study show that online Last Aid courses are feasible and well accepted by citizens. They may reach out to different participant groups to normal Last Aid courses enabling a greater part of the public to participate in public palliative care education. More research on the effects of online Last Aid courses is necessary.

## Figures and Tables

**Table 1:** The Last Aid course contents (actual version updated 2018).

Module Nr.	Topic	Course Content
Module 1	Dying as a normal part of life	Welcome and introductions First Aid and Last Aid What you can do to care The process of dying
Module 2	Planning ahead	Networks of Support Making decisions Medical and ethical aspects Advance care planning Advance Directive Medical Power of Attorney
Module 3	Relieving suffering	Typical problems and symptoms Caring/relieving suffering Nutrition at the end of life How to comfort
Module 4	Final goodbyes	Saying goodbye/final farewell rituals Funeral and various forms of burials Grieving is normal Grief and ways of grieving Questions, Comments

Last Aid—Care for seriously ill and dying people at the end of life.

**Table 2:** Ranking of the online Last Aid course by the participants (*n* = 92).

Question	Very Good	Good	Satisfactory	Enough
What did you think of the course?	74 80%	17 19%	1 1%	0
How did you find the opportunity for participating in discussions?	40 44%	35 38%	15 16%	22%

**Table 3:** Answers about effects of the online Last Aid course (OLAC).

Question	Yes	No
Would you recommend the online Last Aid course to others?	92 100%	0 0%
Do you feel more prepared to encounter death, dying and grief?	90 98%	2 2%
Has the course contributed to preparing you for caring for dying people?	81 88%	11 12%
Do you know how to find information about options for local support in your area?	78 85%	14 15%

## Data Availability

Not applicable.

## Supplementary Materials

The following are available online at https://www.mdpi.com/2227-9032/9/2/172/s1, File S1: English summary of a checklist for Online Last Aid Courses from the guideline estab-lished by the working group.

## Appendix A

**Table A1:** The Online Last Aid Course questionnaire.

**Dear participant of the Online Last Aid Course,** **Last Aid Germany considers itself a learning system. Through your feedback about the Online Last Aid Course, in which you have participated today, using this questionnaire you can contribute to improving the course and adapting it to the needs of the people. We would like to thank you in advance for your support**	
**Please tick a box:**	
What did you think of the course?	
Very good	O
Good	O
Satisfactory	O
Enough	O
How did you find the opportunity for participating in discussions?	
Very good	O
Good	O
Satisfactory	O
Enough	O
Would you recommend the Online Last Aid course to others?	
Yes	O
No	O
Do you feel more prepared to encounter death, dying and grief?	
Yes	O
No	O
Has the course contributed to preparing you for caring for dying people?	
Yes	O
No	O
Do you know how to find information about options for local support in your area?	
Yes	O
No	O
Do you have any other comments or suggestions?—Please write….	
