# Supplementary material for: First Experiences with Online Last Aid Courses for Public Palliative Care Education during the COVID-19 Pandemic
Source: Healthcare (Basel). 2021 Feb 5;9(2):172. doi: 10.3390/healthcare9020172 (PMC7914870; doi:10.3390/healthcare9020172)
Supplement: Supplementary file 1 [file healthcare-09-00172-s001.pdf]

Supplementary File S1. English summary of a checklist for Online Last Aid Courses from the guideline established by the working group.

The summary of the most important suggestions from the guideline can be used as a checklist and is divided in the four headings: Technical Aspects, Preparation, Realization and Postprocessing

Technical Aspects

- Choose an online platform which is stable and which offers the possibility to split-up into small groups
- Prepare yourself, if possible, with two screens (to see both the group and the presentation)
- Good sound (participants forgive minor technical glitches, but do not forgive trouble with sound)
- Experience and practice are required to handle technical problems by yourself (or support by an experienced coach)
- Use of a headset is an option.
- Close the virtual room after starting the session (due to data protection rules and to prevent non-participants from entering the session)
- Provide your phone number (mobile phone number) in the event of technical problems
- Good quality view on the instructors (choose appropriate background and take account of backlighting)

Preparation

- Test your own internet connection and WLAN for stability
- Organize a pre-course meeting, to check the participants tech. and to explain the course process (mainly for technically unexperienced participants)
- Send the participants instructions for use of the platform and support
- Post course material well in advance (texts, addresses, mouth care equipment, swaps etc.)
- At least one of the two instructors should use a second computer to control the presentation and prepare the whiteboard
- Ask the participants to switch off their audio to avoid disturbing noises
- Regulations for a binding registration are necessary
- In general, ask participants to switch on their video (unless there are technical problems like unstable connections)

Realization

- Consider muting participants when entering the session.
- Define rules, consider the use of the chat function.
- Perform a round of introductions to strengthen the personal contact and the relation to the subject. However, keep it short.
- Do not allow video recording (via adjustments)
- Provide a break every 45 min. Conducting an online course is more strenuous than an ordinary course for all participants.
- Prioritize interaction and strive for at least one interaction with the participants within every part.
- Methodical and didactical tips for online courses can be found in “Life aid“
- Integrate the course evaluation into your program. Sending the evaluation afterwards results in reduced return rates.

Postprocessing

- Send confirmation of participation by email.
- Send contact addresses of Hospice- and Palliative Care Services for further questions and concerns.

If you require help in starting Online Last Aid courses you can contact experienced colleagues from the working group. Don’t hesitate to get in touch with Letzte Hilfe Deutschland gUG.
